# Phonetic acquisition in cortical dynamics, a computational approach

**DOI:** 10.1371/journal.pone.0217966

**Published:** 2019-06-07

**Authors:** Dario Dematties, Silvio Rizzi, George K. Thiruvathukal, Alejandro Wainselboim, B. Silvano Zanutto

**Affiliations:** 1 Universidad de Buenos Aires, Facultad de Ingeniería, Instituto de Ingeniería Biomédica, Ciudad Autónoma de Buenos Aires, Argentina; 2 Argonne National Laboratory, Lemont, Illinois, United States of America; 3 Computer Science Department, Loyola University Chicago, Chicago, Illinois, United States of America; 4 Instituto de Biología y Medicina Experimental-CONICET, Ciudad Autónoma de Buenos Aires, Argentina; 5 Instituto de Ciencias Humanas, Sociales y Ambientales, Centro Científico Tecnológico-CONICET, Ciudad de Mendoza, Mendoza, Argentina; Fraunhofer-Institut fur Nachrichtentechnik Heinrich-Hertz-Institut, GERMANY

## Abstract

Many computational theories have been developed to improve artificial phonetic classification performance from linguistic auditory streams. However, less attention has been given to psycholinguistic data and neurophysiological features recently found in cortical tissue. We focus on a context in which basic linguistic units–such as phonemes–are extracted and robustly classified by humans and other animals from complex acoustic streams in speech data. We are especially motivated by the fact that 8-month-old human infants can accomplish segmentation of words from fluent audio streams based exclusively on the statistical relationships between neighboring speech sounds without any kind of supervision. In this paper, we introduce a biologically inspired and fully unsupervised neurocomputational approach that incorporates key neurophysiological and anatomical cortical properties, including columnar organization, spontaneous micro-columnar formation, adaptation to contextual activations and Sparse Distributed Representations (SDRs) produced by means of partial N-Methyl-D-aspartic acid (NMDA) depolarization. Its feature abstraction capabilities show promising phonetic invariance and generalization attributes. Our model improves the performance of a Support Vector Machine (SVM) classifier for monosyllabic, disyllabic and trisyllabic word classification tasks in the presence of environmental disturbances such as white noise, reverberation, and pitch and voice variations. Furthermore, our approach emphasizes potential self-organizing cortical principles achieving improvement without any kind of optimization guidance which could minimize hypothetical loss functions by means of–for example–backpropagation. Thus, our computational model outperforms multiresolution spectro-temporal auditory feature representations using only the statistical sequential structure immerse in the phonotactic rules of the input stream.

## Introduction

It is well known that human beings can reliably discriminate phonemes as well as other linguistic units by categorizing them, despite considerable variability across different speakers with different pitches and prosody. Furthermore, this ability extends to noisy and reverberant environments.

Although such proficiency could in part be attributed to top-down information [1] originated in the grammatical and semantic [2, 3] dimensions present in human language–beyond the phonetic features in the speech signal–trained animals are also able to discriminate phoneme pairs categorically and to generalize in novel situations [4–10]. For instance, cortical activations in naive ferrets revealed the existence of spectro-temporal tuning in Primary Auditory Cortex (A1) with the capacity of supporting discrimination of many American English phonemes [11], even when stimuli were distorted by additive noise and reverberation [12].

It is even more remarkable that an extremely complex task of early language acquisition as is the segmentation of words from fluent speech, is fulfilled by 8-month-old infants based simply on the statistical relationships between neighboring speech sounds [13]. With only 2 minutes of exposure to a continuous speech stream generated by a speech synthesizer, infants showed succesful phonetic acquisition and discrimination. Furthermore, in the training phase there was no acoustic information about word boundaries beyond the statistical structure in the phonotactic rules immerse in the stimuli, and the subjects received no external associative supervision or reinforcement which could have guided or boosted the phonetic acquisition task, which was entirely incidental.

This incidentally acquired invariance in phonetic perception found in mammals must be grounded necessarily in anatomical and neurophysiological characterisitcs of the mammalian cortex. The features we foresee as potentially relevant are brought together in order to pose our computational hypotheses.

### Anatomical and neurophysiological characteristics of mammalian cortex

Linden and Schreiner [14] highlighted that although auditory cortical circuits have some unique characteristics which require special attention, their similarities with other sensory regions–such as visual or somatosensory cortex–turn out to be categorical. First, at the sensory level, the cochlear one-dimensional frequency map could be analogous to the two-dimensional spatial maps which are found in the retina or body surface. Second, the tonotopic maps found in the auditory system could be analogous to the retinotopic and somatotopic organization found in visual and somatosensory cortices, respectively. Frequency tuning curves in the auditory system could correspond to inhibition of spatial surrounding boundaries in visual and somatosensory receptive fields. A correspondence could be drawn between amplitude modulation rate in the auditory system and flicker sensitivity in the visual system, or whisker vibration sensitivity in the somatosensory system. Finally, auditory receptive fields tuned for frequency-sweep, could be analogous to visual and somatosensory motion sensitivity.

Compelling physiological studies have shown that Primary Auditory Cortex (A1) shares common structural characteristics with other sensory cortices. Furthermore, when retinal inputs are routed into the auditory thalamus, auditory cortical cells develop visual response properties such as direction selectivity, orientation preference and complex and simple receptive fields [15–17]. Retinotopic maps, in terms of orientation tuning with lateral connectivity between orientation domains, emerge in superficial layers of the rewired auditory cortex [18, 19].

The above data suggest the existence of neuronal circuitry with similar processing capabilities for different modalities. Consequently, we gather physiological and anatomical characteristics found in cortical tissue in general which we foresee as relevant for phonetic perception invariance and generalization.

**Fig 1:**
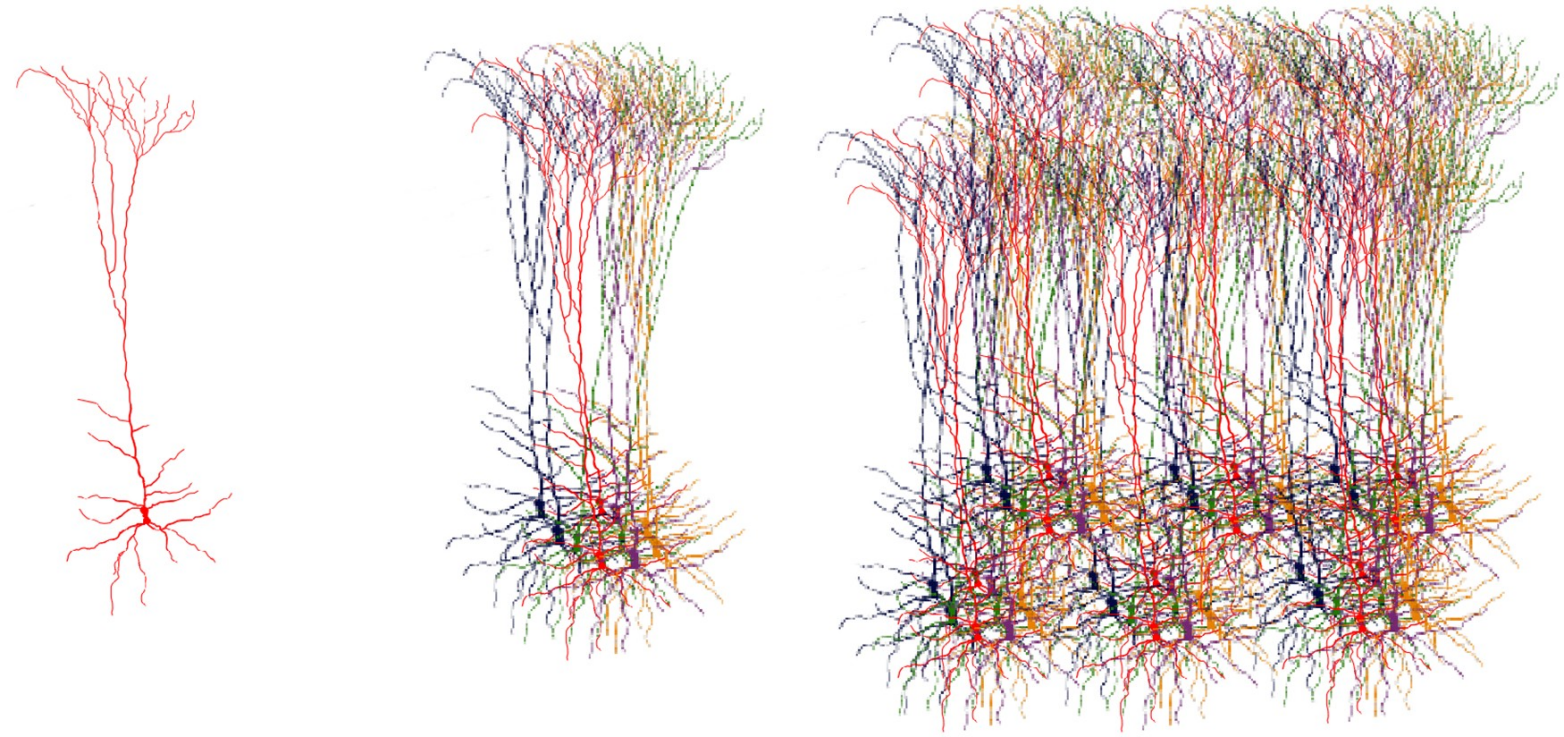
Cortical tissue organization. Left: Pyramidal cell. The most common excitatory neuron in cortical tissue. Center: Cortical mini-column. A cluster on neural cells which responds to stimuli of similar characteristics. Right: Cortical Column. A group of mini-columns with a common receptive field location. Adapted from (Fabuio, Own work, CC BY 4.0, https://commons.wikimedia.org/w/index.php?curid=60707501).

One of the main neuroanatomical features of brain cortex in mammals is that cortical cells are spacially arranged into domains defined by common receptive field locations. These alignments are called Cortical Columns (CCs) [20–23]. Within CCs, cortical mini-columns are clusters of cells which respond to stimuli with similar characteristics (Fig 1). In addition, cortical columns are connected within and between different regions in cortical tissue forming a complex and yet organized connectivity network [24].

One of the functional properties found in many of these networks is adaptation to contextual stimuli [25, 26]. This mechanism is thought to enhance efficiency in the codification of sensory information. For instance, a reduction in the responses to frequent sounds by means of inhibitory networks, may enhance cortical sensitivity to rare sounds that may represent unexpected events [27–29].

Finally, recent findings in neuroscience show that mammalian cortex processes information by means of SDRs [30]. This mechanism allows robust and low-error-rate discrimination of stimuli representations minimizing the neuronal activation during the task in relation to the neural resources available for the representation [31]. Hawkins et al. [32] hypothetize that one of the mechanisms that might be involved in cortical networks in order to achieve SDRs implies the extended depolarization of the soma as the result of independent dendritic NMDA branch activations produced by the excitation of certain number of distal synapses [33, 34].

**Fig 2:**
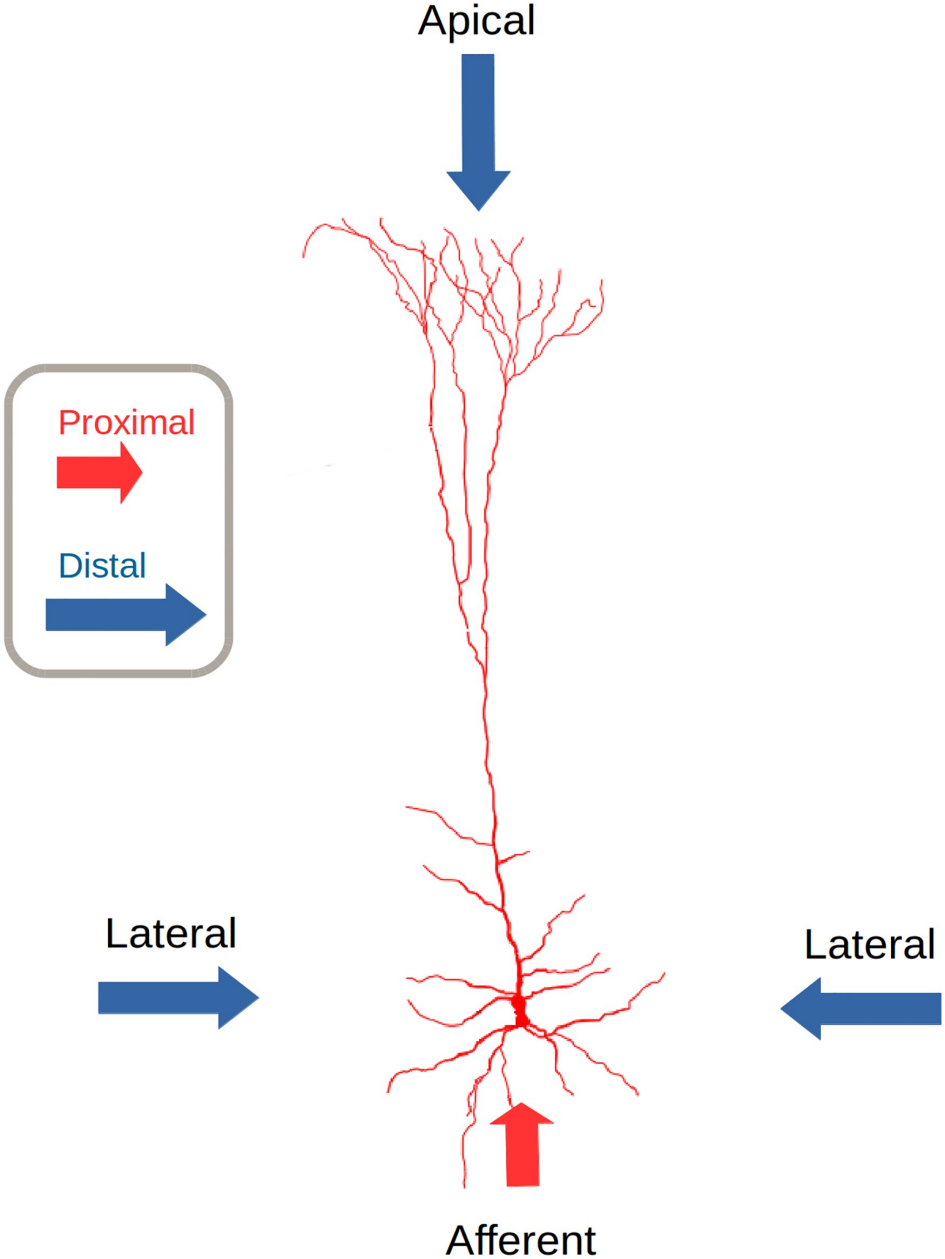
Connectivity profile of a pyramidal neural unit in the Encoder Layer (EL). Proximal connections are formed only by afferent connections from the Multiresolution Spectro-Temporal Sound Analysis (MRSTSA) while distal connections are formed by lateral and apical connections from neighboring columns and from columns in another cortical layer above respectively. The EL is the most important stage in our computational approach while the MRSTSA pre-processes the audio corpora in order to feed the EL. Adapted from (Fabuio—Own work, CC BY 4.0, https://commons.wikimedia.org/w/index.php?curid=60707501).

In the present work the above mentioned anatomical and neurophysiological features of the mammalian cortex are gathered as potentially relevant in order to attain phonetic invariance in the mammalian auditory cortex. Our pyramidal neuron model dissociates proximal from distal dendritic branches (Fig 2). Proximal dendrites act as a homogeneous set receiving only afferent information. Information in proximal dendrites determines a bunch of neural units in a CC which could be activated depending on the previous activations in the same as well as in neighboring CCs.

Distal dendrites receive only lateral and apical information acting as independent detectors. Distal dendritic information pre-activates neural units putting them in a predictive state in order to receive future afferent information.

We test those principles paying special attention to temporal dynamics of speech, which play the most important role in linguistic contrasts [35]. We use a completely unsupervised and biologically-inspired computational model, since we aim to mimic infant incidental phonetic acquisition in whose circumstances no supervision could be justified. Our model produces levels of phonetic classification accuracy similar to those of state-of-the-art deep pattern classification approaches. We therefore propose an alternative path towards addressing phonetic discrimination based on observing structural and functional properties present in the mammalian cortex.

## Materials and methods

### Corpora generation

We generate corpora of 500 words with mono, di and trisyllabic randomly chosen English words from 10 different vocabularies of five words for each syllabic condition using Festival Text to Speech Synthesis [36].

We generate cross synthesizer mark-up-language files with SABLE [37]. In such files, we instruct Festival Text to Speech to generate corpora with 500 words from vocabularies of 5 words uttered by 10 different voices available from the synthesizer.

The organization of the corpora has certain rules and restrictions in order to avoid biases in the training processes. The voices are sequentially chosen (pseudo-randomly) with the restriction that no voice could utter a second time until all the voices had uttered in their turns. Every voice utters two words per turn–in pseudo-random order–and no word is repeated until all the words are used by such voice.

We use two sets of 10 different English speaking voices, each provided by Festival. Set one consisted of 8 male and 2 female voices: cmu_us_fem_cg, cmu_us_gka_cg, cmu_us_ksp_cg, cmu_us_rxr_cg, cmu_us_jmk_cg, cmu_us_rms_cg, cmu_us_slt_cg, cmu_us_jmk_arctic_clunits, cmu_us_rms_arctic_clunits, cmu_us_slt_arctic_clunits. Set two had 5 male and 5 female voices: cmu_us_ahw_cg, cmu_us_aup_cg, cmu_us_axb_cg, cmu_us_eey_cg, cmu_us_awb_cg, cmu_us_bdl_cg, cmu_us_clb_cg, cmu_us_ljm_cg, cmu_us_bdl_arctic_clunits, cmu_us_clb_arctic_clunits.

Every word in the audio file is followed by a silence gap whose time is equivalent to the uttering time of the monosyllabic word *cat*, uttered by the same voice used for the last word. We use the text2wave program provided by Festival in order to generate a wav file from the SABLE file.

We generated all the datasets (audio file corpora) employed in the present research to train the EL and the SVMs and to test the complete Cortical Spectro-Temporal Model (CSTM). This folder includes a set of 840 corpora which are distributed in 2 corpora for each configuration organized by 2 sets of synthesized voices, 3 syllabic conditions and 10 vocabularies all distributed in 6 acoustic variants, beyond the original version of the corpora. The 6 acoustic variants corresponds to: two levels of white noise (19.8 dB and 13.8 dB Signal to Noise Ratio (SNR) average Root Mean Square (RMS) power rate), two levels of reverberation (Reveberation-Time 60 dB (RT-60) value of 0.61 seconds and 1.78 seconds) and variations of pitch on both directions (from E to G and from E to C).

### Computational model

We propose a computational approach called CSTM, which simulates a patch of cortical tissue and incorporates columnar organization, spontaneous micro-columnar formation, partial NMDA depolarization and adaptation to contextual activations. We simulate pyramidal cells with proximal connections from afferent dendritic branches and distal connections from lateral dendritic branches. Similar afferent stimuli activate clusters of neurons with proximal physical locations in a CC in the same way that afferent information activates the mini columns found in cortical tissue.

Afferent information activates different clusters of units in a CC establishing a first and raw approximation of the phonetic features abstracted from the input auditory stream. Our model fine-tunes such raw features by means of previous contextual activations produced in the same and/or in neighboring CCs. Such contextual information is sent to each CC by means of lateral distal dendritic branches which work as independent processing elements in a cell. Current activation in such dendritic elements will affect the way in which cells receives future afferent information.

Novel computational theories have posited a feasible explanation about the role of distal synapses related to NMDA phenomenon [32] by combining it with SDRs [31]. In our model, we adopt a similar approach to the one in [32] in which current activation patterns produce partial depolarization of certain cells by means of distal dendritic branch connections. A state of partial depolarization is sustained in time in some cells within future afferently excited clusters of neurons. Partially depolarized cells fire in advance with respect to other cells in the excited clusters, thereby preventing other cells from firing by means of proximal lateral GABAergic inhibition, obtaining in this way, SDRs.

In addition, we simulate the growth of distal dendritic branch synapses by means of Spike-timing dependent plasticity (STDP) mechanisms together with homeostatic regulations. In this way, distal synapses will be established only among pyramidal cells with sequential patterns of activation. Afferently excited clusters which do not have partially depolarized cells, will fire together producing a Massive Firing Event (MFE) (lack of inhibition) as a response to a prediction fault (unexpected sequential stimuli in the stream of data); otherwise they will respond with normal firing events (inhibition and therefore SDRs) when the sequential stimulus is correctly predicted.

SDRs exhibit interesting mathematical properties which give them high noise rejection and fault tolerance [38]. These are typical characteristics in cortical tissue where individual cells are far from 100% reliable and the cells die and regenerate continuously. To simulate this phenomenon, we incorporate stochastic characteristics by which neural cells inside afferently activated clusters are chosen to be active by a discrete distribution whose probabilities are determined by the afferent excitability of individual cells during training.

Hence, the evolution of our network does not predetermine a neuron to fire but biases its probability of doing so during training. Additionally and under specific conditions, afferent dendritic arborizations activate themselves at random with levels whose boundary values are established by learning.

It has been shown that overfitting–a phenomenon in which a statistical model describes random error or noise instead of the underlying relationship–is greatly reduced by stochastic properties in training procedures applied to neural networks (dropout) [39].

In order to produce the inputs from auditory streams we base on MRSTSA [40]. In our software implementation, we primarily follow its cortical section rather than its sub-cortical counterpart, incorporating different neurophysiological phenomena found in A1 [41] such as symmetry [42], bandwidth [43], and frequency modulation selectivity [42, 44, 45].

The CSTM consists of two parts: The Multiresolution Spectro-Temporal Sound Analysis (MRSTSA) layer and the Encoder Layer (EL).

The algorithm MRSTSA, which processes the sound waves to feed inputs to the EL, is a technique inspired by Chi T. et al. [40]. In their work, accumulating experimental findings from the central auditory system were exploited demonstrating its applications in the objective evaluation of speech intelligibility. As the authors pointed out, the model was not biophysical in spirit, but rather it abstracted from the physiological data an interpretation which was likely to be relevant in the design of sound engineering systems. In our MRSTSA implementation, we follow main guidelines from the higher cortical representations developed in [40].

The EL converts a multidimensional array of real numbers into a multidimensional Sparse Distributed Representation (SDR). This stage is composed by a set of Self Organizing Maps (SOMs) [46, 47] and incorporates neurophysiological phenomena such as columnar organization, afferent spontaneous micro-columnar formation, proximal and distal dendritic arborization, lateral intercolumn interaction by means of independent dendritic NMDA branch activations, MFEs with contextual stimulus adaptation, proximal lateral intracolumn inhibition, Long-Term Potentiation (LTP), Long-Term Depression (LTD), STDP and distal synaptic homeostatic regulations.

#### Multiresolution Spectro-Temporal Sound Analysis (MRSTSA)

As mentioned above, Chi T. et al. [40] developed a computational model of auditory analysis inspired by psychoacoustical and neurophysiological findings in early and central stages of the auditory system.

The original algorithm has a subcortical and a cortical stage. For the subcortical stage, first an affine wavelet transform of the acoustic signal represents the spectral analysis performed by the cochlear filter bank. Second, the cochlear filter outputs are transduced into auditory-nerve patterns by a hair cell stage consisting of a high-pass filter, a nonlinear compression and a membrane leakage low-pass filter. Third, a first-order derivative with respect to the tonotopic axis followed by a half-wave rectifier simulates the action of a lateral inhibitory network postulated to exist in the cochlear nucleus, which effectively enhances the frequency selectivity of the cochlear filter bank. The final output of this stage is obtained by integrating over a short window, with time constant of 8 ms, mimicking the further loss of phase locking observed in the midbrain.

The cortical stage mimics aspects of the responses of higher central auditory stages, especially A1. Functionally, this stage estimates the spectral and temporal modulation content of the auditory spectrogram. It does so computationally via a bank of filters that are selective to different spectrotemporal modulation parameters that range from slow to fast rates temporally, and from narrow to broad scales spectrally. The Spectro-Temporal Receptive Fields (STRFs) of these filters are also centered at different frequencies along the tonotopic axis.

**Fig 3:**
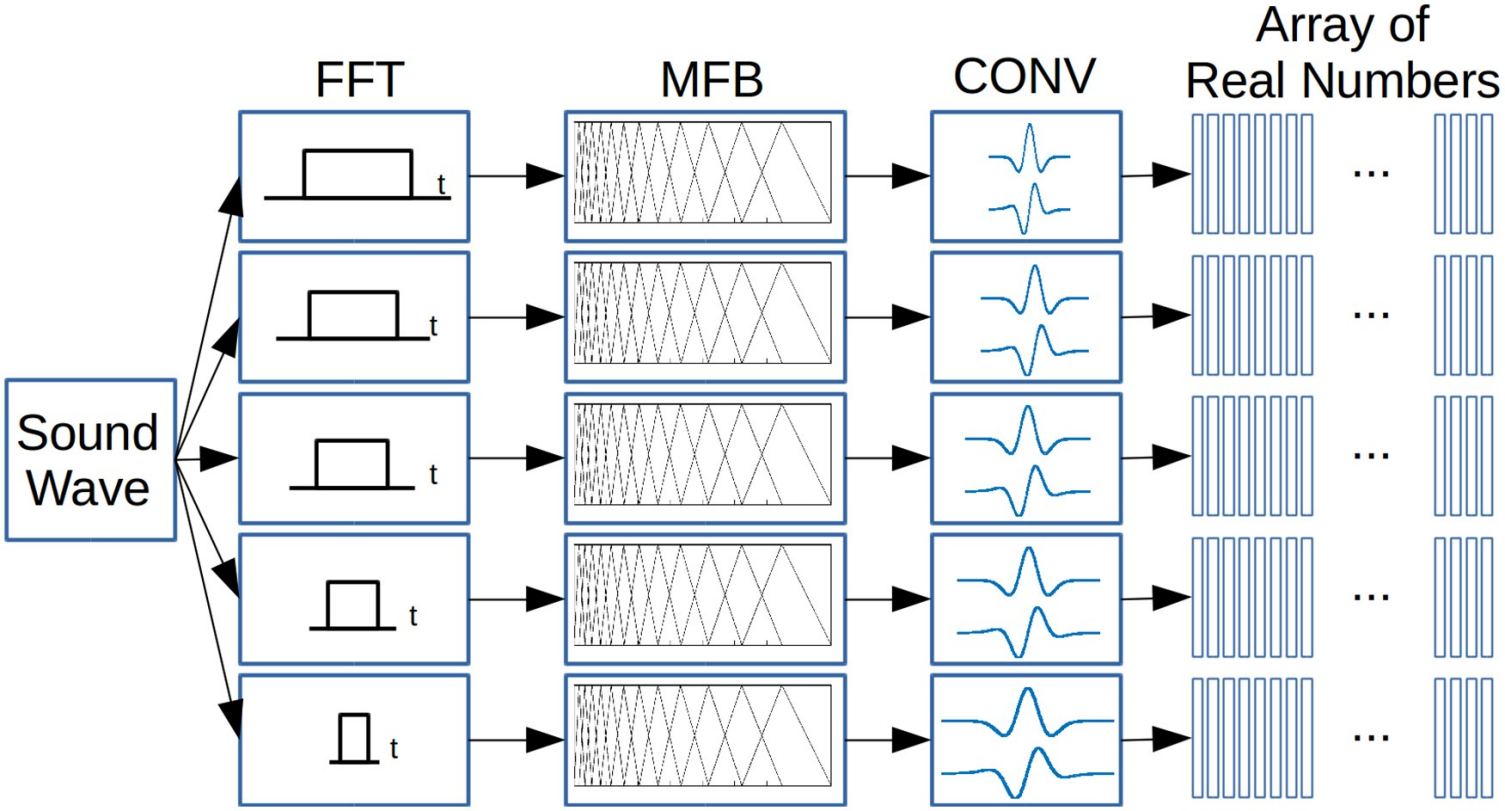
Multiresolution Spectro-Temporal Sound Analysis (MRSTSA) algorithm. Sound waves are processed by FFTs with different time windows, then each spectrum is processed by a Mel Filter Bank (MFB) and each resolution is convolved with a complex signal with a different coefficient. Finally, each filter coefficient is obtained computing the modulus from the convolution and then applying an automatic gain control.

In the present work, since we aim to integrate neurophysiological properties–mainly centered in cortical features–we followed the main guidelines in the implementation of the cortical section of such model. As shown in Fig 3, we implemented the initial stage in our model with the application of FFT to the audio vector with a different sample window for each resolution. We then extracted the power spectral density from each resolution. In this way we obtained a multiresolution spectral analysis of the audio signal, with high spectral and low temporal resolution for wider sample windows and vice versa. Such different time windows in the FFT, incorporated–at the same time–leakage low-pass filters with a time constant for each resolution accounting for decrease of phase-locking in the auditory nerve. We then applied a Mel Filter Bank (MFB) with 128 elements to each spectrum in order to represent the spectral analysis performed by the cochlear filter bank. Then, we convolved each resolution obtained in the last step along its tonotopic axis with a complex multiresolution function whose real part was a symmetric Mexican hat function and its imaginary part was its antisymmetric Hilbert transform. With this strategy we simulate the phenomena of symmetry [42], bandwidth [43] and frequency modulation selectivity [42, 44, 45] found in A1 and incorporated in the original algorithms [41].

We obtained the magnitude of each convolution and applied normalization to each time window as a mean of automatic gain control in order to prioritize the information delivered by the spectral configuration and not the absolute values delivered by the filters.

By means of this constraint we account for the mechanical and chemical properties of hair cells in the mammalian inner ear which constitute a transduction mechanism that appears to adapt to recent stimulus history in a way that can affect its gain [48–50]. We decided to be conservative, not including sound intensity dimension but just the shape of the filter responses.

#### Encoder Layer (EL)

The EL is responsible for generating SDRs from the inputs delivered by the MRSTSA stage described in the previous section and from the activation history in its own CCs.

The EL simulates a patch of cortical tissue called Cortical Layer (CL)using an n-dimensional array of complex structures called Complex Self-Organizing Maps (CSOMs) that simulate CCs in the brain.

**Fig 4:**
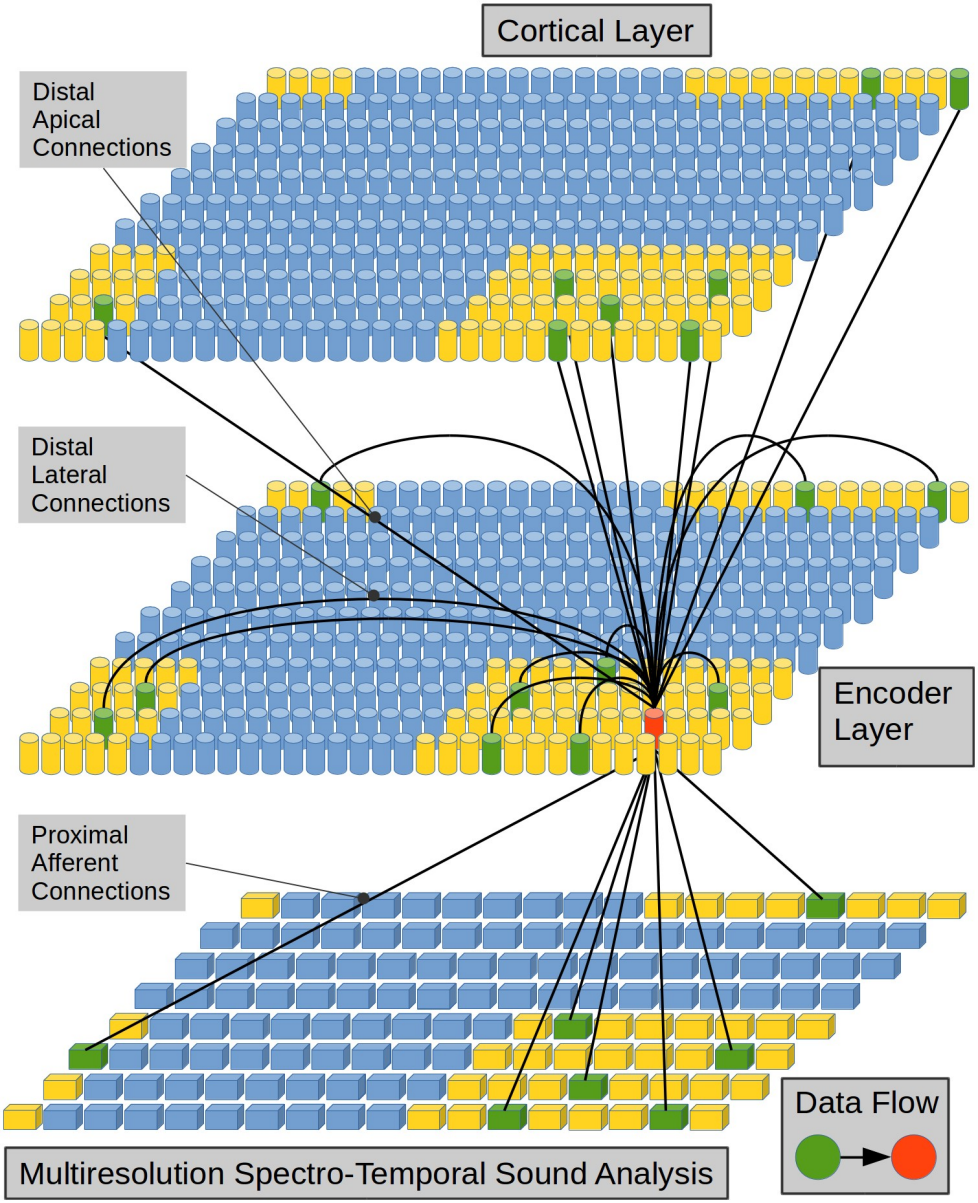
Connection scheme for a cortical column in the Encoder Layer. Each cylinder in the EL and in the CL represents a CC in neural tissue. Each prism in the MRSTSA represents a real valued variable. This is a visualization of a CC (in red) and its three receptive fields (in yellow). The receptive field of a CC is an array that defines a set of CCs with which such column could be connected. The receptive field of a CC on the MRSTSA determines an array of real valued variables with which such column could be connected. A subset of CCs in a receptive field (in green) represents the CCs that are really connected with the CC in red. A similar scenario could be described for the green prisms on the MRSTSA. The size, wrap-around property and percentage of established links (in green) inside a receptive field are tunable parameters for the model. In this work, only lateral connections have been implemented since in the current implementation there are no upper cortical layers from which to bring apical connections.

Each CC in the EL is connected to the MRSTSA below by means of afferent connections. It is also connected to neighboring CCs–including possibly itself–in the EL by means of lateral connections and to CCs from other CLs above by means of apical connections. Such connection scheme is shown in Fig 4.

Both lateral and apical are feedback connections that constitute contextual information channels. These channels put the current afferent excitation under the context of previous activations. Such connections damp the activity of some units allowing only the precise activations of specific neural units in an afferently excited CC. Such precise activations match the sequential paradigms learned by the network.

Recent findings in neuroscience [51] support the idea that feedback could potentially enhance visual representations in time and space damping the activity of certain cells while allowing the activations of others which agree with their predictions.

In the present work we only implement lateral connections since there are no upper layers from which to bring apical information in the present implementation.

Each cell unit in a CC has two types of dendritic branches; proximal and distal. Proximal and distal dendritic branches lead to proximal and distal connections in a cell unit respectively. Proximal and distal connections produce different effects on a neural unit’s plasticity and activation. Neural units in the EL simulates pyramidal cells in cortical tissue in the brain. Fig 2 shows the connectivity profile in such units.

**Fig 5:**
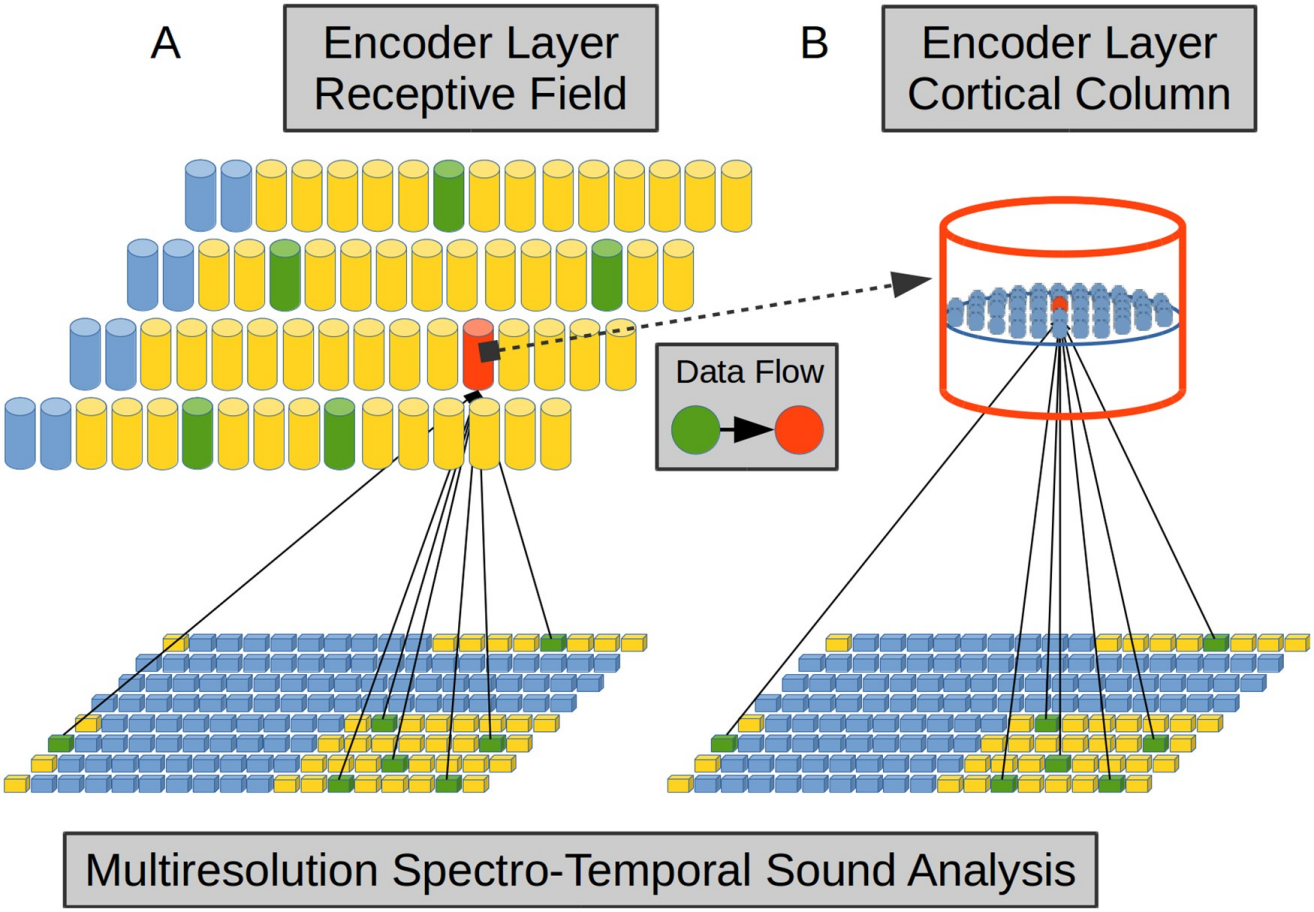
Encoder Layer (EL) proximal connections. Each CC in the EL–exemplified here in red–has its receptive field over the MRSTSA–in yellow. (A) A set of MRSTSA components–in green inside the receptive field–is randomly chosen to be connected with such CC. (B) Each neural unit in such CC is connected with the same set of MRSTSA components.

In reference to proximal dendritic connections in the EL, each neural unit in a CC has the same set of proximal connections to the MRSTSA (Fig 5). Such connections constitute a multidimensional space of real numbers. In order to acquire the statistical distribution in such multidimensional real space we use a multidimensional SOM in each cortical column (Alg. 1).

**Algorithm 1** Plasticity in Proximal Synapses. Self Organizing Map (SOM) algorithm.

1: given an input vector, find the nearest unit to such input vector in the input space

2: move such unit towards the input vector in the input space (the magnitude of such movement depends on the learning rate)

3: also move neighbor units to the nearest one towards the input vector (the magnitude of such movement depends on the learning rate and on a neighborhood measure over the topology of the network of units)

A SOM is an unsupervised clustering algorithm which distributes a continuous multidimensional distribution in a discrete multidimensional distribution of units [46, 47]. In this way we ended up with an array of units of *m* dimensions in which each unit represents a set of vectors from the continuous distribution in an input space of *n* dimensions. Generally, *m* < *n* in order to reduce the dimensionality in the discrete representation. We added such restriction in our columnar algorithm.

In the SOM algorithm, each input vector has to be completely determined. In our case, several elements in the inputs from the MRSTSA could be null, and considering such null inputs could inpair learning. Hence, each input vector could not have the information of each of its components available. We incorporated a stochastic mechanism in the EL in order to deal with such situation. We made each afferent connection to learn statistical boundaries from its corresponding input. We establish a minimum-maximum margin in each proximal connection in the EL. Such margin is consistent with the statistical distribution in the history of its corresponding input. When an afferent input is undetermined, in a context in which some afferent inputs have available information, the EL chooses the value in the undetermined input randomly between the boundaries learned for such input.

We call our implementation of the SOM algorithm, Static Self-Organizing Map (SSOM). The SSOM algorithm accounts for proximal lateral intra-column interaction, LTP and LTD. It also dissociates proximal dendritic inputs from distal dendrites, since it modifies proximal connections following the statistical distribution from the MRSTSA independently of the units that fire in such CC. This independence in the plasticity of the proximal dendritic inputs is supported by the property found in cortical tissue by means of which there is dendritic plasticity in the context of partial depolarization of the soma [52]–that is, without an Action Potential (AP).

The term *static* comes from the fact that the patterns learned from proximal afferent dendrites do not account for the contextual history in the dynamic evolution of the algorithm.

**Fig 6:**
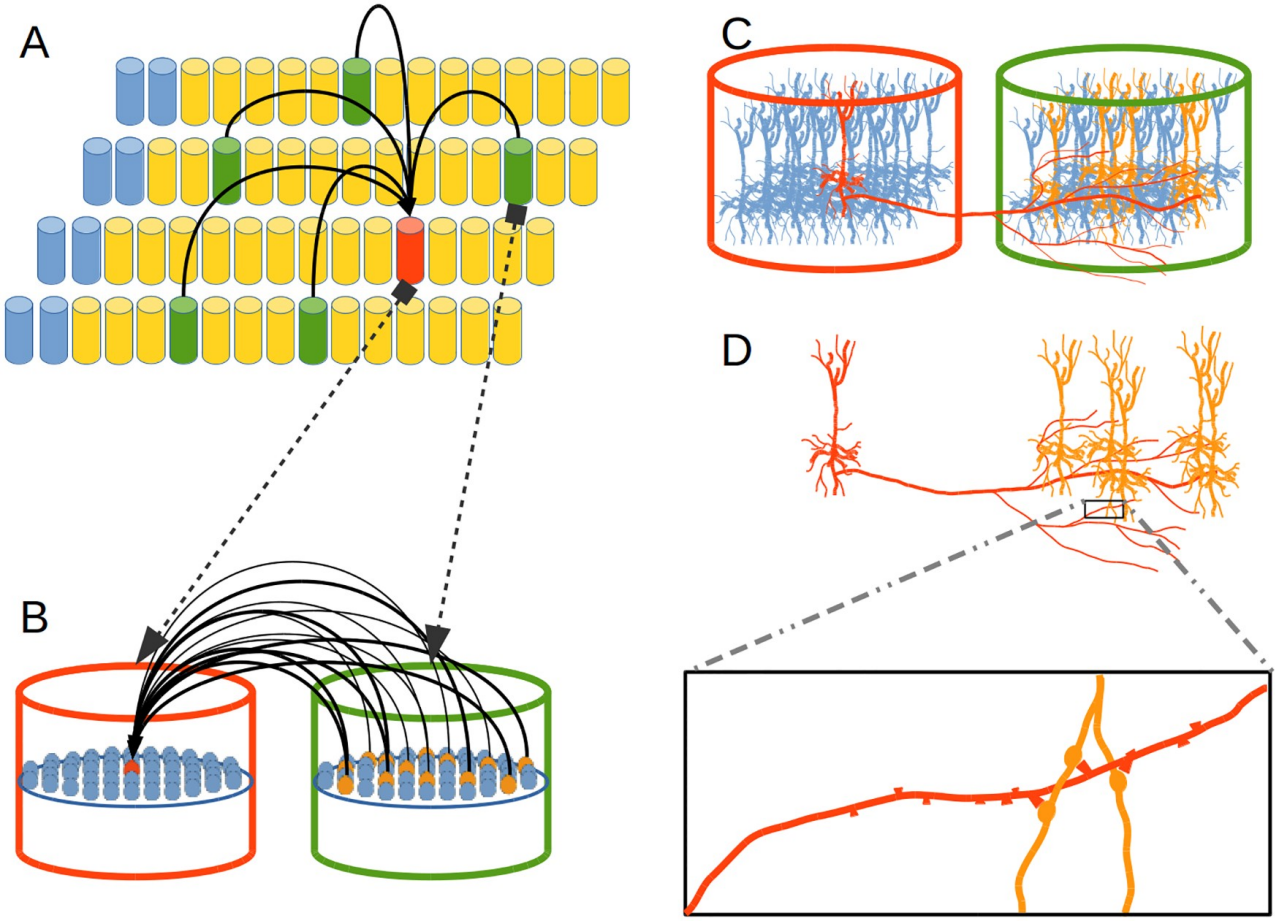
Distal dendrite connections. (A) Distal dendritic branches from neighboring CCs inside the receptive field of a CC in the EL. A distal dendritic branch between the red CC and a green CC means that every neural unit in the red CC is linked with a different subset of neural units in the green CC by means of potential connections. (B) Potential connections in a dendritic branch which link a neural unit in the red CC with a subset of neural units in a green CC. The subset of potential connections comes from a percentage of neural units inside the green CC. Such percentage is a tunable parameter for the CC. (C) A distal dendritic branch between a pyramidal cell in a CC and a sub-set of pyramidal cells in a neighboring CC inside its receptive field in the EL. (D) Physical proximity of a dendritic branch from the red cell to axonal branches from yellow cells constitutes potential connections which could prosper becoming in established synapses depending on the sequential activity among cells.

In terms of distal dendritic branches, each CC in the EL is connected to other CCs–in green in Fig 4–by means of such branches inside the receptive fields–in yellow in Fig 4–from the same EL and from another CL above. Each link between the red CC and a green CC–Fig 6A–symbolizes the fact that each cell unit in the red CC is linked with a different subset of cell units in the green CC–Fig 6B.

Such links in Fig 6A, represent dendritic branches in neural tissue and we call each connection in Fig 6B, potential connection. Potential connections represent synapses in the dendritic branch. A cell unit inside the red CC ends up with as many dendritic branches as green CCs inside its receptive field (Fig 6A).

The term *potential connection* is used, because it describes a pair of neural units linked by its physical location and dendritic and axonal disposition in cortical tissue (Fig 6C). However, an effective connectivity between such neurons will depend upon their sequential pattern of activation which will establish developed synapses between them. If two neural units–a red one and a yellow one in Fig 6D–are linked by means of a distal potential connection–produced by a synapse between a distal dendritic branch from the red one and an axonal branch from the yellow one–such connection will grow only if there is a sequential activation of the red cell after an activation of the yellow cell, in two consecutive time steps. If such phenomenon does not repeat itself over time, such synapse will decrease its strength with respect to other synapses in the dendritic branch in the red cell in Fig 6D. A simultaneous activation in both neural units–the red one and the yellow one in Fig 6D–will decrease the strength in such potential connection.

We implemented distal dendritic synaptic plasticity mechanisms by means of an algorithm called Dynamic Self-Organizing Map (DSOM) (Alg. 2). The learning mechanisms implemented on such algorithm simulate neurophysiological phenomena such as STDP, and homeostatic regulation plasticity in the synaptic strength regulation in distal dendritic branches.

**Algorithm 2** Plasticity in Distal Synapses. This algorithm accounts for Spiketiming dependent plasticity (STDP) and homeostatic regulation phenomenon in distal dendritic synapses.

1: **for** every active unit in this cortical column **do**

2:  **for** every dendrite in this active unit **do**

3:   increment all the synapses–in this dendrite–potentially connected to units which were active in the last time step

4:  **end for**

5: **end for**

6: **for** every active unit in this cortical column **do**

7:  **for** every dendrite in this active unit **do**

8:   decrement all the synapses–in this dendrite–potentially connected to units which are active in this time step

9:  **end for**

10: **end for**

11: **if** updated step reaches certain value **then**

12:  **for** every unit in this cortical column **do**

13:   **for** every dendrite in this unit **do**

14:    **if** the sum of the synapses in this dendrite is greater than one **then**

15:     normalize all synapses in this dendrite

16:    **end if**

17:   **end for**

18:  **end for**

19:  updated step = 0

20: **end if**

21: updated step++

Basically, Alg. 2 updates all distal axonal-dendritic synapses, incrementing them always that pre and post-synaptic neural units become active in consecutive time steps, and decrementing them when they become active at the same time step. Occasionally, all the synaptic weights belonging to the same dendrite are normalized every certain number of time steps. This normalization is repeated for all neural units and for all dendritic branches in each neural unit whose sum of synaptic weights is beyond unity.

**Fig 7:**
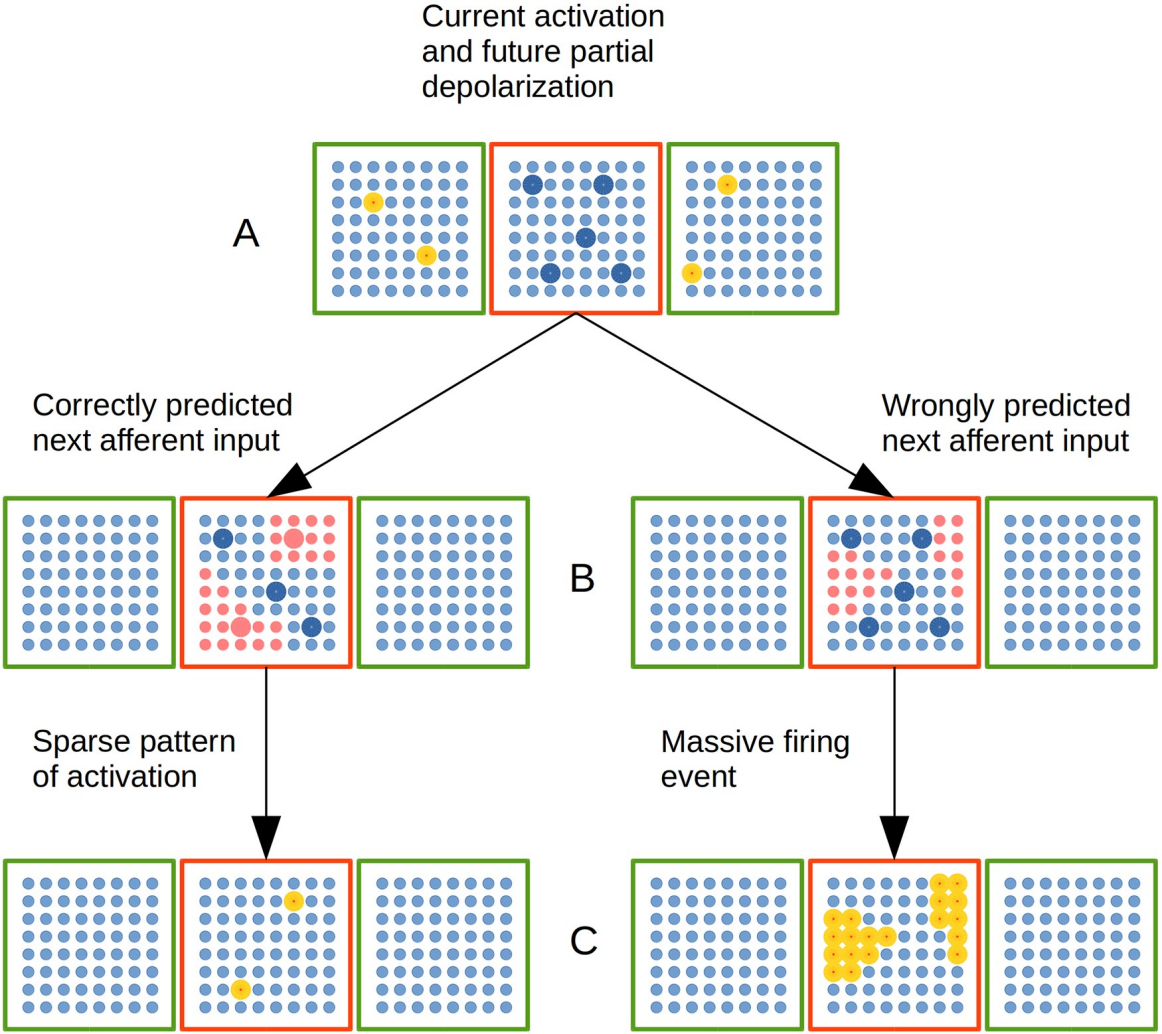
Dynamic cellular activation in a CC in the EL. A red cortical column is linked with two green cortical columns by means of distal dendrites. (A) Cellular activation in green CCs–highlighted yellow cells–puts neural units in red CC in a partially depolarized–predictive state highlighted in blue. (B) Cluster of neural cells activated by afferent inputs. Left: A substantial amount of partially depolarized cells are in the afferently excited cellular clusters. Right: There is no substantial amount of partially depolarized cells inside afferently excited cellular clusters. (C) CC with active cellular units highlighted in yellow. Left: Sparse pattern of cellular activation. Right: Massive pattern of activation.

Finally, in reference to the activation rules of neural units inside a CC in the EL, first a group of cell units in a CC is partially depolarized by distal connections among such neural units and cell units activated in the previous time step in the EL–Fig 7A. That is, neural units activated in time step *t* = 0 in the EL, will partially depolarize a set of neural units in time step *t* = 1 in such CC, by means of distal–lateral and apical–dendritic branch synapses established by learning in the DSOM algorithm.

Second, afferent proximal connections from MRSTSA will tend to depolarize certain clusters of units in such CC in time step *t* = 1–Fig 7B. The tentative depolarization is produced by the inputs from the MRSTSA with proximal synapses established by learning in the SSOM algorithm. Such group of neural units are randomly chosen from a discrete distribution whose probabilities are established by the state of excitation in afferent inputs.

If a sufficient number of partially depolarized units are in the set of afferently excited units, such partially depolarized units will fire previously in the group–Fig 7B left. Those units–which fire before–prevent neighboring units in the excited clusters from firing, hyperpolarizing them by means of lateral inhibitory connections in the column.

Partial depolarization states put cell units in a predictive state generated by the activations produced in the EL in previous time steps. That is, lateral and apical activation in previous time steps constitute a context in which current afferent inputs are received.

From the group of units that tend to be depolarized by current afferent inputs from the MRSTSA, only a reduced sub-set of those units are likely to fire in the previous contextual firing history in the EL–Fig 7C left.

In case there is no context, that is, if not enough units normally depolarized by afferent inputs are partially depolarized by previous–lateral and apical–activations–Fig 7B right–, all units in the afferent excited clusters will be active, covering more hypotheses for next inputs–Fig 7C right.

Such activation mechanism is depicted in Alg. 3. In Alg. 3 (Part 1) a ranking is established among neural units–inside a CC–in terms of its afferent excitability, given the afferent inputs (lines 1 and 2). The *number of afferently excited units* refers to the maximum number of units that can be activated by the afferent input in a CC and *minimum number of active units* refers to the number of units that will be active in a CC if a SDR is achieved as a result of optimal prediction (lines 3 and 4 respectively).

**Algorithm 3** Units activation (Part 1). This algorithm establishes the activation rules in a CSOM object.

1: distances = given an input vector find the euclidean distance each unit has to such input in the input space from proximal afferent synapses

2: ranking = sort indexes from the smallest to the largest distances

3: number of afferently excited units = proximal activation percentage*number of units

4: minimum number of active units = (1-sparsity)*number of units

5: **if** randomness is disabled **then**

6:  excited units = gets the first *number of afferently excited units* elements from ranking

7: **else**

8:  excited units = gets *number of afferently excited units* random indexes from distances with probabilities determined by the relative reciprocal of the distances element values

9: **end if**

10: **for** unit = 0 **to** unit = number of units **do**

11:  auxiliary = 0

12:  **for** dendrite = 0 **to** dendrite = number of distal dendrites **do**

13:   dendrite accumulator = 0

14:   **for** active unit = 0 **to** active unit = number of linked active units **do**

15:    potential index = find the first coincident index in potential connections[dendrite][unit] with linking units[dendrite][active unit]

16:    **if** there exist coincidence **then**

17:     dendrite accumulator += dynamic synapses[dendrite][unit][potential index]

18:    **end if**

19:   **end for**

20:   **if** dendrite accumulator > 100*DISTAL_SYNAPTIC_THRESHOLD **then**

21:    auxiliary++

22:   **end if**

23:  **end for**

24:  total responses[unit] += auxiliary

25: **end for**

26: updated distances = element wise quotient between distances and total responses

27: updated ranking = sort indexes from the smallest to the largest updated distances

If randomness is enabled, *number of afferently excited units* units is chosen at random by means of a discrete distribution whose probabilities are the afferent excitation of each unit. If randomness is disabled, *number of afferently excited units* first units are chosen from the ranking of afferently excited units (lines 5 to 9).

From line 10 to 25 each neural unit accumulates distal–lateral and apical–excitation in order to determine its partial depolarization from units which were active in the previous time step. For each neural unit in a CC, for each distal dendrite in such unit and for each active unit in such distal dendrite the algorithm looks for coincidences between some potential connection in such distal dendrite in the neural unit and the active unit in such distal dendrite. That is, in line 15, the algorithm asks if there is coincidence between some potential connection in this distal dendrite inside the unit and the neural unit activated in the previous time step in the CC linked by such distal dendrite. If there is coincidence, the value of the synaptic weight in such potential connection is accumulated in a dendrite accumulator. After all active units are examined for this dendrite, if the dendrite accumulator is greater than certain threshold, such dendrite is considered active and the total response of the unit is incremented in one.

Each neural unit ends up with an excitation value due to its distal dendrites. The unit distances vector is element- wise divided by distal dendritic excitations vector to get the updated distances and an updated ranking of the units (lines 26 and 27). In this way, units with more distal excitation will decrease its distance more and will be put in a more favorable place in the ranking in order to be activated.

In Alg. 3 (Part 2) the minimum updated distance is found in the group of afferently excited units. Then, a set of units–inside the group of afferently excited units–is identified which have such minimum updated distance. While the number of identified units is less than *minimum number of active units*, the next minimum updated distance is found in the group of afferently excited units and a new set of units–inside the group of afferently excited units–is identified which have such next minimum updated distance. This new set is added to the previous one until the number of units in this accumulative set is greater than or equal to the minimum number of active units.

**Algorithm 3** Units activation (Part 2). This algorithm establishes the activation rules in a CSOM object.

1: new distances = get the updated distances elements whose indexes are in excited units

2: new minimum distance = get the minimum element from new distances

3: minimum indexes = get indexes from updated distances vector whose values are equal to new minimum distance

4: apt to be active = get the coincident indexes between excited units and minimum indexes

5: erase from new distances vector, all the elements whose value is equal to new minimum distance

6: **while** number of elements in apt to be active vector < *minimum number of active units* and new distances has at least one element **do**

7:  new minimum distance = get the minimum element from new distances

8:  minimum indexes = get indexes from updated distances vector whose values are equal to new minimum distances

9:  partial apt to be active = get the coincident indexes between excited units and minimum indexes

10:  incorporate partial apt to be active elements into apt to be active vector

11:  erase from new distances vector, all the elements whose value is equal to new minimum distance

12: **end while**

13: **if** ENABLE_RANDOM_BEHAVIOUR **then**

14:  shuffle apt to be active vector

15: **end if**

16: **for** number = 0 **to** number = number of apt to be active elements **do**

17:  incorporate to output the excited units[apt to be active[number]]

18: **end for**

19: **return** output

The functional result of Alg. 3 is that there must be a sufficient amount of–partially and previously depolarized–neural units inside the afferently activated cluster of units in order to get a SDR pattern of activation. Otherwise, the CC will end up with a massive activation pattern, a Massive Firing Event (MFE) in which more than a *minimum number of active units* will be active. In the case of the occurrence of a MFE, the synaptic plasticity is modulated in order to form stronger synapses of those neural units activated during such event.

Each neural unit in a CC establishes its state of partial depolarization based on the contribution from distal dendritic branches from lateral and apical connections. A dendritic branch will contribute to the partial depolarization of the soma in such cell only if such dendritic branch exceeds an activation threshold by means of the contribution from its individual synapses in the context of the patterns of activation in the previous time step.

This mechanism has compelling sequential properties [32], which have already been applied in the classification of artificially generated sequential data [53]. We apply such mechanism in the DSOM algorithm by adding the contribution of synapses–in a dendritic branch–whose connections are linked with cells that were active in the previous time step in the EL.

#### Implementation

Regarding MRSTSA, we apply FFT to the audio files with a sample period of 8 milliseconds. We use the FFTW package [54, 55] with time windows of 8, 16, 32, 64 and 128 milliseconds in order to obtain a multiresolution power spectral analysis of the signal. In addition, we apply the Mel Filter-Bank technique with 128 filters to each spectral resolution and convolve such filters along their tonotopic axis. For the convolution, we use a multiresolution complex function whose real part is a Mexican hat function and its imaginary part is the corresponding Mexican hat Hilbert transformation.

The function coefficients are 10 for the 8 ms time window, 8 for the 16 ms time window, 6 for the 32 ms time window, 4 for the 64 ms time window and 2 for the 128 ms time window. We then compute the magnitude from the convolution and normalize in each time step. By means of this procedure we obtain from the audio file a multiresolution spectro-temporal response composed by an array of 128 columns–one column per filter–and 5 rows–one row per resolution–with real numbers which range from 0 to 1, for each time step.

In reference to the EL, we implement an EL with 225 CSOMs arranged in a two-dimensional array of 15 by 15 CCs. Each CC is automatically distributed using individual locations along its afferent inputs in a uniform way. Each CC receives afferent information by means of two-dimensional afferent receptive fields of 5 by 227 filters centered at individual locations over the MRSTSA. We enable the wraparound property in order to make each receptive field span the entire MRSTSA array. We also instruct each column to receive only 31 inputs, which is a minor percentage of such receptive field. Individual afferent inputs for each CC are chosen randomly in the EL initialization.

For this model instance we implement only distal lateral dendritic branches since there are no more CLs from which to bring information through apical dendritic branches. We configure each CC to have a lateral receptive field with 9 by 9 neighboring CCs and to receive information from 72 of the 81 CCs in the receptive field–a 90% of the receptive field.

Each CC is composed of a two-dimensional array with 15 by 15 (225) neural units and each unit in a column could be potentially connected with only 6 neural units from each linked neighboring column. That is, each neural unit in a CC ends up with 72 lateral dendritic branches with 6 potential connections each (432 distal potential synapses per cellular unit). Such potential synapses are randomly chosen for each neural cell and for each dendritic branch in the cell during the Encoder initialization procedure. The EL consists of 50625 cellular units with 1569375 proximal synapses and 21870000 distal synapses. Such specifications state the number of free parameters of the model, but it is important to highlight that distal synapses represent potential connections from which only a small percentage has a significant synaptic weight as to be considered as an established connection. Weak synapses are periodically pruned by means of homeostatic processes in the network leaving distal dendrites with a sparse connectivity in the receptive fields. Typical sparseness in such connectivity matrices could exceed the 90%.

We train the EL using a 500 word corpora generated by the procedure described in section Corpora generation. The training procedure consists of 4 stages and for each stage the EL receives the same corpus 4 times.

During each learning stage, certain parameters–such as the learning rates in proximal and distal synapses and the lateral intra-column interaction–are exponentially and progressively decreased from an initial value, which also decreases for each successive stage. An additional stage is executed with the learning parameters fixed.

The sparsity in the activation for each CC is 99% (just 2 neural units out of 225 could be active for normal activation events). On the other hand, the afferent excitation affects 10% of the units inside the clusters in each CC (22 neural units, which could be activated in case of a MFE; Fig 7).

**Fig 8:**
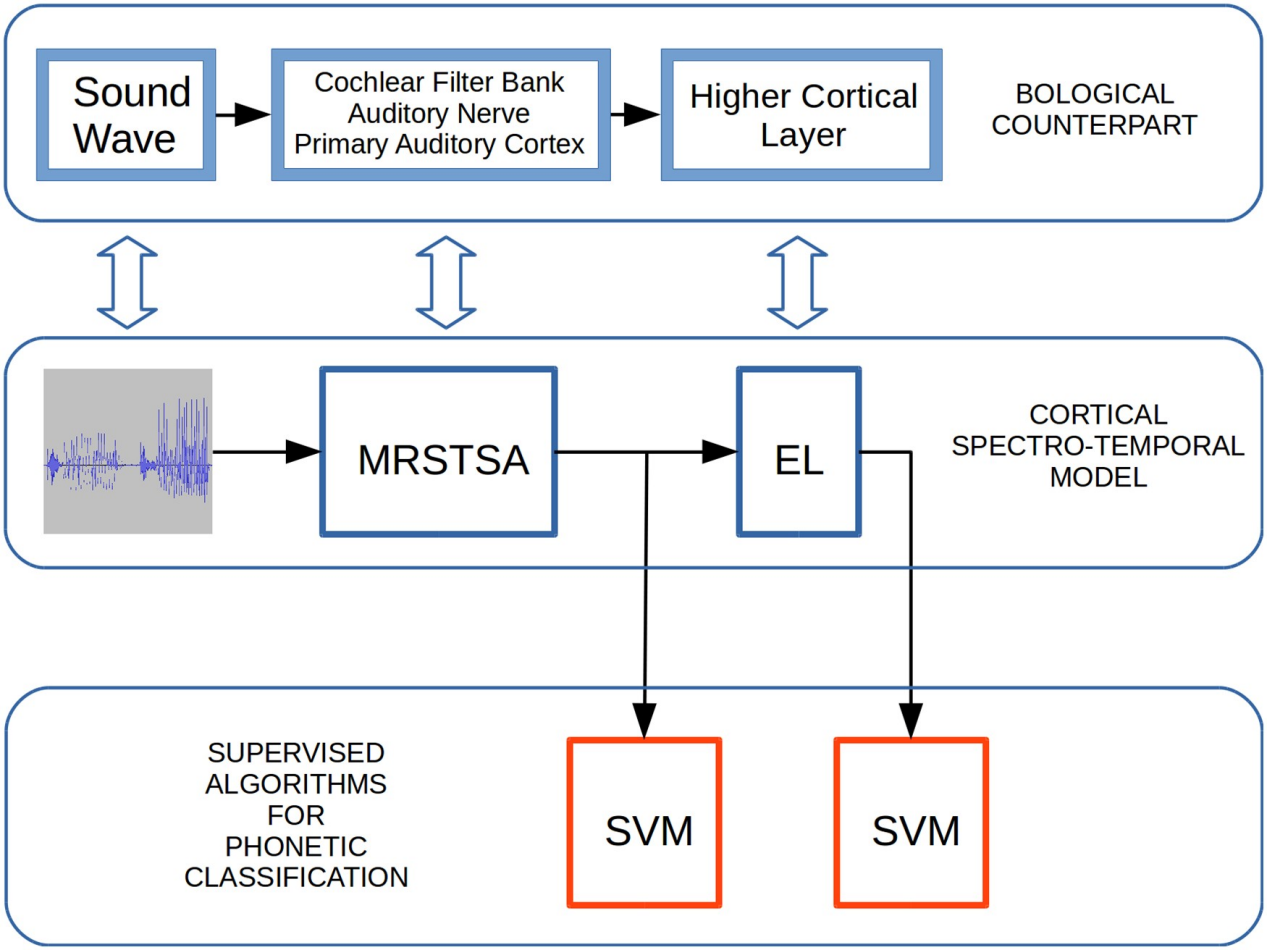
Experimental setup to test word classification task performances. Sound waves are processed by the MRSTSA algorithm. The outputs from the MRSTSA are processed by the EL. Word classification tasks are performed on both outputs by the SVM algorithm. Each section in the CSTM has its biological counterpart.

In regards to SVM classification, we use supervision by means of the SVM classification method, receiving the outputs from each algorithm [56, 57]. We do this to test the invariance properties in the phonetic features abstracted by the EL in comparison with the phonetic features abstracted by the MRSTSA, (Fig 8).

We use the silent temporal gaps between consecutive words in the MRSTSA outputs in order to introduce marks to detect the beginning and end of each word.

We then produce a vector per word in the corpus summing the activity in the MRSTSA as well as in the EL between consecutive marks and use such vectors to train both classifiers (the one receiving outputs from the MRSTSA and the one receiving outputs from the EL).

Afterwards, we scale the vectors–as the Library for Support Vector Machine (LIBSVM) documentation suggests–so as to improve the classification performance. We train and test the SVM classifiers using 5-fold cross-validation and configure them to use a linear kernel with one parameter *C* which we swept to find the best trained model for each classifier.

#### Experiments

In the present work, we studied the level of invariance in the phonetic features abstracted by the EL, by means of comparing such representations with the multiresolution spectro-temporal auditory features returned by the MRSTSA algorithm. To this end, we evaluated the features returned by each algorithm in different word classification tasks. In order to asses word classification performance in each algorithm, we used the SVM technique–section Computational model–with the experimental setup depicted in Fig 8.

In the experimental procedure we first trained 10 different ELs–section Computational model–for each syllabic condition. Such ELs were trained using the voices in set one and the corpora were generated by the method described in section Corpora generation. Afterwards, we processed the same corpora with the corresponding ELs in inference mode. In such mode, the ELs processed the information with their learning properties disabled. In this manner, during inference, the ELs did not modify its synapses and just returned patterns of activation in response to the stimuli they received. We then used the outputs from the MRSTSA and the ELs in inference mode to train the SVM classifiers with the procedure depicted in section Implementation. The average cross validation training performances are shown in Table 1.

**Table 1:** SVM 5-fold cross validation training results.

	MRSTSA	Encoder Layer
Monosyllabic Words	99.4%	99.52%
Disyllabic Words	99.3%	99.48%
Trisyllabic Words	99.5%	99.58%

In a second stage, we ran the ELs in inference mode again, but this time we used different corpora generated using the same voices and manipulated with several types of acoustic variants (white noise, reverberation and pitch variations), generated using the Audacity software. We also ran the ELs in inference mode using corpora generated with different voices (set two voices in section Corpora generation). We tested the performances of the–already trained–classifiers in the presence of the features returned by the algorithms in response to the corpora affected by the acoustic variants which we introduced to the new corpora by means of Audacity [58]. The acoustic variants introduced to the new corpora included white noise, reverberation and pitch variations. We also tested the classifier performances in the presence of the features returned by the algorithms in response to the new corpora generated with a different set of voices.

## Results

**Fig 9:**
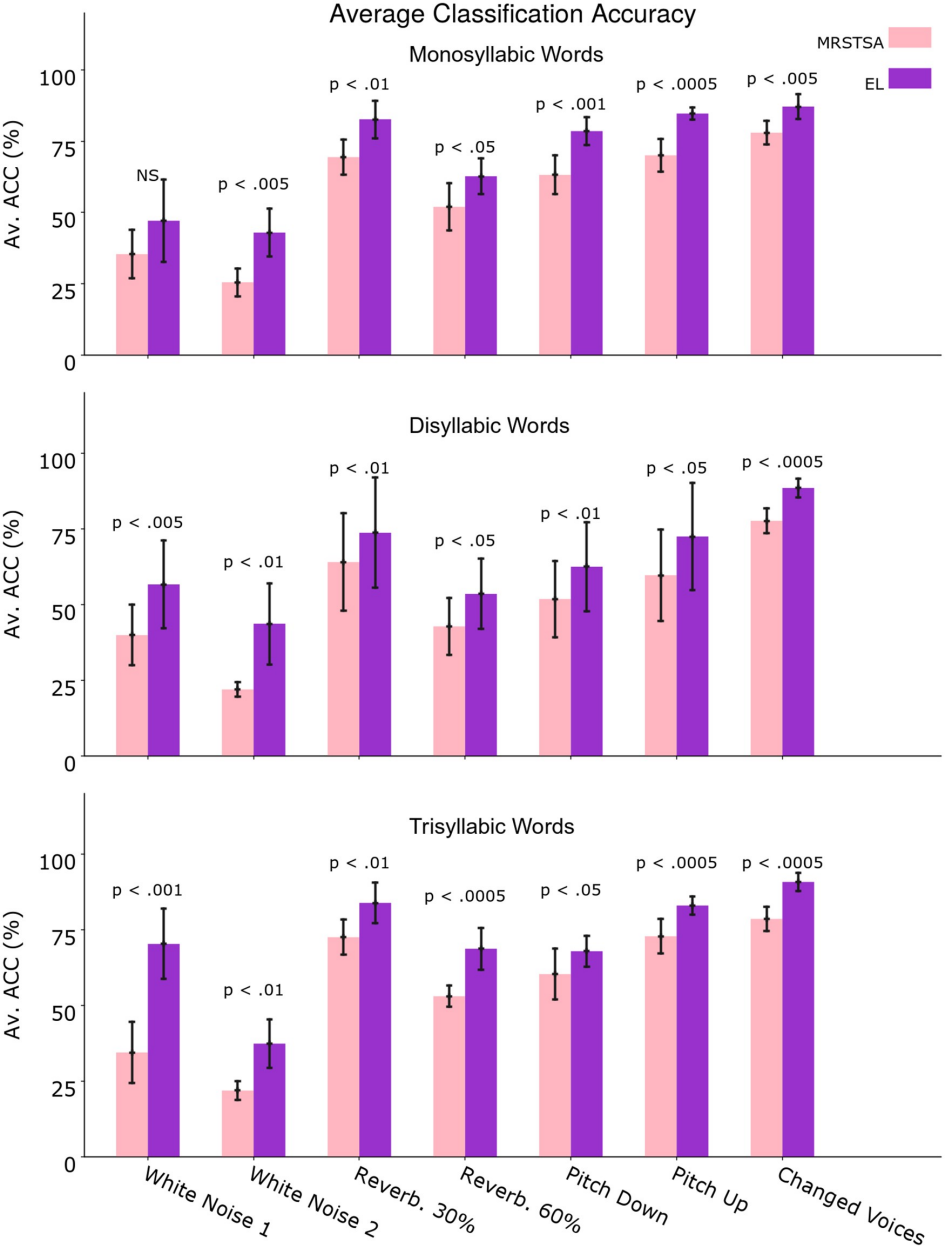
MRSTSA and EL average classification accuracies against different acoustic variants introduced to the signals for monosyllabic, disyllabic and trisyllabic words. White Noise 1 determines a SNR average RMS power rate of 19.8 dB while White Noise 2 13.8 dB. Reverberation 30% determines a RT-60 value of 0.61 seconds while Reverberation 60% determines a RT-60 value of 1.78 seconds. Pitch Up determines a pitch move from E to G, while Pitch Down determines a pitch move from E to C. Changed Voices corresponds to corpora generated using a different set of voices from the one used to train the ELs and the classifiers. Error bars depict 95% Confidence Interval values. The *p* values correspond to two-tailed paired t-tests and NS stands for Not Statistically Significant.

The classification performances are shown in Fig 9.

Regarding white noise, we introduced additive white noise to the corpora signals with signal-noise average RMS power rate of 19.9 dB (White Noise 1) and 13.8 dB (White Noise 2). In terms of reverberation, we modified the corpora signals by means of RT-60 values of 0.61 seconds (Reverberation 30%) and 1.78 seconds (Reverberation 60%). RT-60 Is the time that a signal takes to decrease its amplitude to 60 dBs under its initial value. As regards pitch variations, we modified the corpora signals pitch in +20% (from E to G) (Pitch Up) and in–20% (from E to C) (Pitch Down). We also used corpora generated with different voices from the ones used to train the ELs and the SVMs.

Fig 9 shows a 5-way word classification accuracy for mono, di and trisyllabic word corpora affected by white noise, reverberation and pitch and voice variations. As can be seen in the figures, the EL outperforms the MRSTSA in all cases. Such behavior persists for multisyllabic words.

We performed two-tailed paired t-tests for 10 different corpora generated from 10 different vocabularies–section Corpora generation. As can be seen in Fig 9–except for monosyllabic words with White Noise 1 (*p* < 0.22)–there was Statistical Significance for all conditions considering (*p* < 0.05).

Given that we conducted 7 t-tests for each independent word classification task (i.e. mono, di and trisyllabic words), we performed Holm–Bonferroni corrections with a correction factor of 7 in order to reduce the probability of type I and type II errors in the context of the different experimental conditions [59]. By means of such corrections we confirmed the statistical significance for all the cases showed in Fig 9.

**Fig 10:**
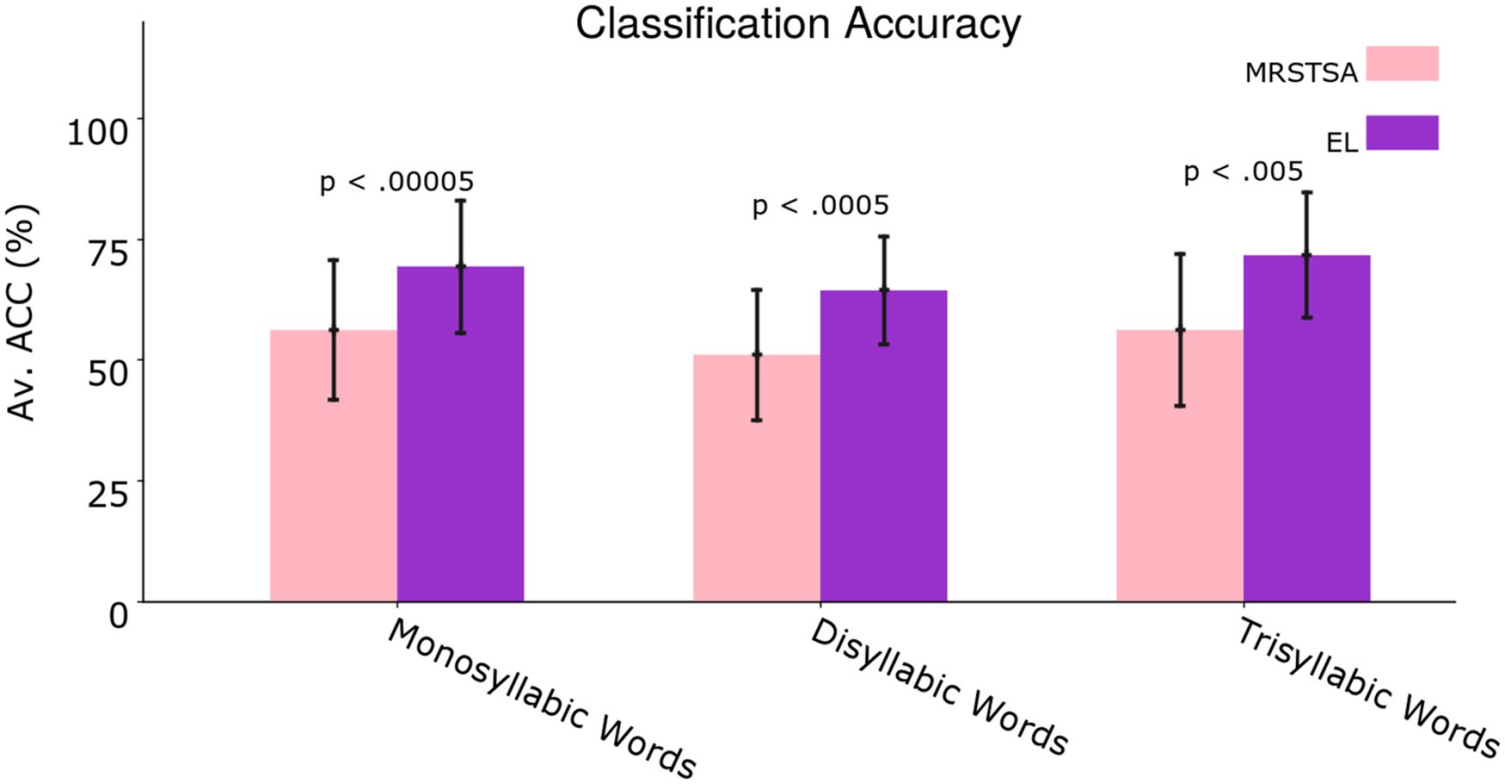
Average classification accuracies across all acoustic variants for mono, di and trisyllabic words. Error bars depict 95% Confidence Interval values. The *p* values correspond to two-tailed paired t-tests.

Fig 10 shows average classification accuracies across all acoustic variants for mono, di and trisyllabic words. In this case, we also performed two-tailed paired t-tests, but this time for 7 different acoustic variant conditions. As can be seen in the figure, all performed tests are statistically significant and the encoder layer clearly shows a sustained superiority across words with different number of syllables.

## Discussion

Results obtained in the present work support the computational hypotheses posed in our modeling approach in order to mimic incidental phonetic invariance and generalization. Some of these hypotheses have already been explained in terms of their properties [32], but more specifically in terms of their sequence learning capabilities [53]. Nevertheless, there are no precedents of such neurophysiological features tested in word classification tasks as the ones carried out here, in which phonotactic rules are acquired without the application of optimization procedures such as backpropagating errors by means of gradient descent. In addition, our approach presents substantial differences in terms of feature algorithmic implementation. In the present work, distal synapses make continuous individual contributions and our anatomical micro-columnar organization acquires its physiological behavior spontaneously from learning. We also tested such features in a realization with hundreds of cortical columns each combining several micro-columns with stochastic afferent activation whose future implementations are intended to explode large-scale simulations in leadership supercomputers.

Computational models have been previously developed to understand how phonetic categories are acquired [60]. The goal in these works has been mainly to explain relevant aspects of phonetic acquisition, without details about how the brain might provide such computations. Lee et al. (2009), employed unsupervised feature learning for audio classification with Convolutional Deep Belief Networkss (CDBNs) [61]. The authors tested classification performance of a model with two layers in a 39-way phone classification accuracy task on the test data Acoustic-Phonetic Continuous Speech Corpus (TIMIT) for various numbers of training sentences. The first layer never outperformed the Mel Frequency Cepstral Coefficients (MFCC) algorithm that was used as input for the network. Furthermore, such work did not report the second layer performance since it could not outperform the first one. The maximum performance reported for the first layer was 64.4% vs. a performance of 79.6% for the Mel Frequency Cepstral Coefficients (MFCC). It was just possible to report a performance of 80.3% by means of combining both, the Mel Frequency Cepstral Coefficients (MFCC) and the first layer in the CDBN.

In a more recent work, the capacity of Deep Maxout Networks (DMNs)–a modification of Deep Neural Networks (DNNs) feed-forward architecture that uses a max-out activation function–to handle environmental noise was investigated into different broad phonetic classes and for different noise conditions [62]. In such experiments–with the exception of fricatives phonemes for 15 dB SNR Street Noise–accuracy never exceeded 70%. Furthermore, performance was seriously impaired in the presence of 15 dB SNR white noise, resulting in classification accuracy well below 60% in all cases.

In the present work, we reported classification performances of–for example–90.8% on the EL vs. 78.58% on the MRSTSA for trisyllabic words against Changed Voices (i.e. voices never “heard” by the EL during training; Fig 9), we also reported a performance above 70% on the EL vs. a performance below 35% on the MRSTSA for trisyllabic words against 19.8 dB SNR white noise and performances well above 40% for mono and disyllabic words against 13.8 dB SNR white noise (Fig 9). We also reported that the EL outperformed the MRSTSA for all the test conditions and that this behavior was sustained through different number of syllables in the words by means of statistical significance tests (Fig 10).

Although this is a compelling scenario, we have to be cautious since we cannot ignore important experimental differences from previous research results. First, our training material was very different from that found in previous works. We used corpora generated by synthesized voices instead of standardized TIMIT corpora. Our main aim was to mimic early phonetic acquisition in humans, which is incidentally accomplished by infants [13]. Given the high quality of the voices synthesized by Festival Text to Speech [36] and its flexibility in order to compose different kind of corpora–even with words that do not exist in any language–we considered that this was an appropriate initial experimental procedure to test our approach in a context of incidentally acquired phonotactic rules. Second, we pursued multisyllabic words classification tasks in contrast to the phone classification experiments carried out in previous research, since we mainly aimed to test the dynamic sequential capability of our model to acquire the phonotactic rules behind the training vocabularies. Finally, we reported results on 5-way classification tasks vs. performance on 39-way classification tasks in [61]. On the one hand, this last difference may have acted in favour of our approach considering that it is easier to classify one category among 5 than one among 39. On the other hand, it is important to highlight that previous works have had more extended training material with more vocabularies, more speakers, etc. In our case, we presented more difficult training conditions, since our model was trained with 500 words from a vocabulary of just 5 words uttered by 10 voices. Despite the small sample size, the performance obtained by our neurocomputational model exhibits a significant level of phonetic generalization with the capacity to acquire phonotactic rules and to generalize to novel environmental contexts. This is a much more biologically accurate scenario than those settled by other approaches, in which models are trained using millions of examples. Our model therefore mimics experimental results which show that 8-month-old infants acquire the phonotactic rules immersed in auditory input streams with only 2 minutes of exposure (180 words in total, from a vocabulary of four three-syllable words) [13].

We are aware that more tests–in different scenarios–with different and standardized corpora (such as TIMIT) will be needed to analyze the capacities of our approach more deeply. Nevertheless, our main objective in the present work was to assess the sequential phonetic invariance exhibited by the EL under strictly controlled experimental conditions in which we precisely knew the levels of noise, reverberation and pitch variations with which the stimulus was affected. The EL training material included only the original corpora with 500 words, but more importantly, the EL was never exposed–during learning–to the disturbances used to test its classification performance. The experimental profile applied in this work (Fig 8) makes it clear that the EL is completely unsupervised and that all supervision is limited to the SVM algorithm. Furthermore, the EL does not optimize its synaptic weight updates using gradient descent backpropagating errors from arbitrarily inserted loss functions. This is a fundamental point to demonstrate the biological plausibility of our implementation since phonotactic constraints in a human language are learned incidentally [63, 64] and therefore, no supervision could be supported under such behavioral circumstances.

In future researches, emergent dynamic properties could arise from the addition of subsequent cortical layers–beyond the Encoder Layer (EL)–in the processing pipeline of this model. In this way we will be able to implement backward distal apical dendrites, which will bring context through an unsupervised hierarchical implementation. Even though such feedback could be beneficial in an incidental phonetic acquisition context, modeling adult phonetic competence could possibly require the implementation of more complex hypotheses in our model. The incorporation of biologically accurate cost functions in order to feed back any kind of activation error would require precise biological hypotheses. Should those errors be scalar signals (reinforcement mechanisms) or should they be vectors (supervised mechanisms)? Should they come from the same modality or should they be gathered from a different one? Should they vary across different cortical patches or should they vary during temporal development? [65]. A supervised mechanism assisted from different cortical areas in a multimodal fashion could be a biologically accurate hypothesis, since it has been shown that iconic gestures boost speech comprehension under adverse listening conditions [66]. Furthermore, functional connectivity across different cortical areas facilitates speech comprehension when the intelligibility of the speech signal is reduced [67]. Yet, beyond the cost functions hypotheses, it is also important to determine the algorithms used to feed back activation errors. Regardless of the fact that gradient descent is–at first glance–too complex to be implemented by cortical tissue, several studies support the idea that credit assignment–the ultimate goal of backpropagation–could be a phenomenon present in the brain [68]. Furthermore, in [69] the authors presented a mechanism that performs backpropagation relaxing the backward connectivity architecture and assigning blame by multiplying errors by random synaptic weights. Apart from the above, there is no proof that the brain implements gradient descent as it is implemented in current DNNs, thus novel strategies–with more biological plausibility–could arise from the scientific community in the future.

Future versions of the model will increase biological plausibility by increasing the number of cells per CC with massively scaling High Performance Computing (HPC) simulations and using a Growing Self Organizing Map (GSOM) per CC in order to incorporate neural resource recruitment specialization in each CC depending on the statistical dispersion of its stimuli [70]. For instance, a four-dimensional array of neural units can be employed to simulate cortical columns of approximately 34,000 cells. In this way, thousands of cortical columns can be organized in multidimensional arrays. Using a leadership-class supercomputer (e.g. resources from the Top 500 computing list, top500.org), and assuming one cortical column per compute node with 64 cores, we could be running 527 neural units per thread in a CPU. Furthermore, configuring a three-dimensional array of 1000 cortical columns per cortical layer, a model of 4 layers could be running on approximately 256,000 threads. Such simulations could allow us to leverage phonotactic acquisition as well as phonetic generalization capacities beyond the levels reported in this paper.

## Conclusion

We show via a computational simulation that our cortical model leverages the performance in word classification tasks under specific environmental conditions (e.g., white noise and reverberation) and for certain acoustic variants applied to the auditory stimuli (e.g., pitch and voice variations). The model acquires the phonotactic rules in the input, without any kind of supervised or reinforced optimization procedure, taking only advantage of auto-organized algorithmic properties. We also show effectiveness in classifying multisyllabic words, which suggests that our implementation of neurophysiological predictive dynamics plus stochastic sparse patterns of activation outperforms the MRSTSA algorithm in terms of phonotactic sequential invariance for disturbances applied to the audio signal. Most importantly, the present model–based on current neurophysiological and neuroanatomical data of the human auditory pathway–is able to mimic incidental phonetic acquisition observed in human infants, which is a key mechanism involved during early language learning. Increasing the models complexity (by addition of further cortical layers), could allow the model to replicate further mechanisms involved during human language acquisition, such as inferential learning or prediction generation. In addition, neurophysiological and anatomical properties in our model could be considered potentially relevant to the design of artificial intelligence systems and may achieve higher levels of phonetic invariance and generalization than the ones achieved by current deep learning architectures.
